# Field data sets for seagrass biophysical properties for the Eastern Banks, Moreton Bay, Australia, 2004–2014

**DOI:** 10.1038/sdata.2015.40

**Published:** 2015-08-04

**Authors:** Chris M. Roelfsema, Eva M. Kovacs, Stuart R. Phinn

**Affiliations:** 1 Remote Sensing Research Centre, School of Geography, Planning and Environmental Management, University of Queensland, Queensland 4072, Australia

**Keywords:** Biodiversity, Ocean sciences, Environmental sciences, Plant ecology

## Abstract

This paper describes seagrass species and percentage cover point-based field data sets derived from georeferenced photo transects. Annually or biannually over a ten year period (2004–2014) data sets were collected using 30–50 transects, 500–800 m in length distributed across a 142 km^2^ shallow, clear water seagrass habitat, the Eastern Banks, Moreton Bay, Australia. Each of the eight data sets include seagrass property information derived from approximately 3000 georeferenced, downward looking photographs captured at 2–4 m intervals along the transects. Photographs were manually interpreted to estimate seagrass species composition and percentage cover (Coral Point Count excel; CPCe). Understanding seagrass biology, ecology and dynamics for scientific and management purposes requires point-based data on species composition and cover. This data set, and the methods used to derive it are a globally unique example for seagrass ecological applications. It provides the basis for multiple further studies at this site, regional to global comparative studies, and, for the design of similar monitoring programs elsewhere.

## Background & Summary

This paper presents a unique point based data set that was collected over the period 2004 to 2014 and which describes the spatial and temporal distribution of seagrass species and their horizontally-projected percentage cover for the Eastern Banks, a shallow 142 km^2^ area of Moreton Bay, Australia. Individual data sets were acquired for different research projects, but the collection methods were consistent throughout. The data sets were used to create and validate remote sensing image-based benthic habitat maps (e.g. seagrass species composition, percentage cover and biomass). The initial field data collection design was planned to acquire sufficient field data to describe the spatial distribution and variability of seagrass properties across the study site.

The data sets described in this study were integrated with satellite imagery as calibration and validation data to generate and verify maps of seagrass properties using a variety of remote sensing mapping approaches^1–7^, and several of these maps are now accessible online^8–12^. It is common practice when mapping the environment using remote sensing imagery, to use training (calibration) data to transform the image into a map of surface properties using a multivariate statistical clustering algorithm. Reference (validation) data are subsequently used to determine the accuracy of the output maps^13^. Therefore, field data must be representative of all the features to be mapped. And ideally, field data collection should coincide with satellite image acquisition to avoid seasonal influences (within one month for this study).

The initial maps created were driven by a need for local government agencies to have updated seagrass maps that could be used to assess the state of the local resources. Temporal maps have since been used to understand the spatial dynamics of seagrass species and percentage cover at the study site, and, to enable the evaluation of changes in meadow properties. The seagrass maps generated, therefore, aide in the protection and conservation of this region. These data sets may also be used to measure seagrass biomass, understand species inter-relationships, and, help to measure or model actual and assumed impacts on seagrass meadows as a result of sea level rise, increased pollution or increased sea temperatures.

Other data sets exist that describe seagrass properties (species, percentage cover and biomass) however, their areal coverage and repeat cycle are limited. Seagrass Watch is the most well-known data collection program, and involves a global citizen science-based monitoring program with over 300 study regions in 17 countries^14^. One of those regions is Moreton Bay (1,500 km^2^) in Australia, where Seagrass Watch has 50 sites^15^, of which three are located within the Eastern banks, the focus of this paper. Researchers have also collected data from specific locations in Moreton Bay to characterise flood events. This sampling included three sites at five locations surveyed over the autumn, winter and summer seasons^16^. Therefore, compared to the data set described in this paper, limited data points for different seasons are available and accessible, and, they do not: (1) cover a large extent, consistently, that can be used for monitoring and modelling, or, (2) use the same method over time.

The point data sets presented in this paper could be used for further understanding of seagrass distribution e.g. landscape or seascape dynamics (meadows or component patches (patch metrics)); biomass; species distribution; leaf area index, area growth/attrition; etc). Seagrass biomass, for instance, was modelled for each individual data point using a relationship between biomass derived from approximately 100 seagrass cores, and, species and percentage cover from the data points^17^. Furthermore, detailed analyses of the complete data set and the associated remote sensing based maps^3^ may permit a greater understanding of the persistence and/or dynamics of seagrass in response to environmental and anthropological impacts. For example, when used in conjunction with existing historical water quality data sets^18^, seagrass penetration may be determined. To this degree, these seagrass data seta are invaluable.

## Methods

**Figure 1:**
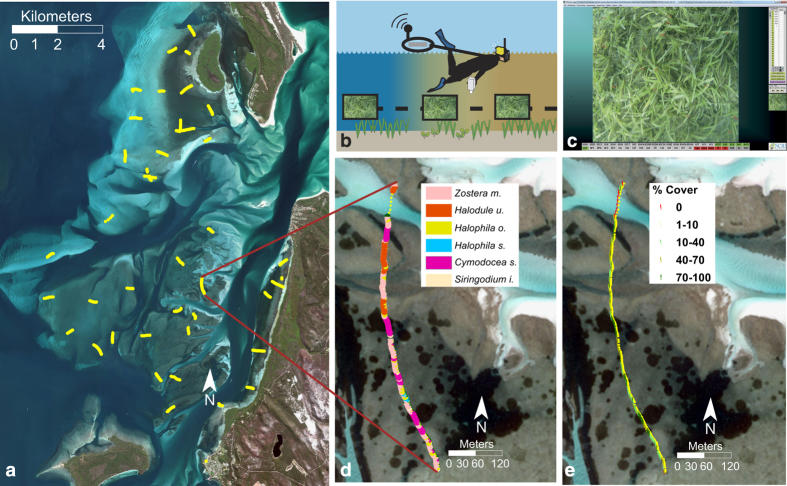
The Eastern Banks seagrass meadows, Moreton Bay, Australia. (**a**) Location of transects on the Eastern Banks; (**b**) conceptualisation of snorkeller-based georeferenced photograph transect surveys; (**c**) screen shot of cover type labelling from photo analysis using Coral Point Count Excel^22^, (**d**) benthic composition showing seagrass species and (**e**) percentage horizontal project seagrass cover for each of the photographs along the transect.

The point based data in this study was collected for the Eastern Banks, Moreton Bay Australia. Photos were analysed for the substrate types, algae and/or seagrass species known to be present on the banks (Figure 1). The seagrass species classes used were: *Halophila spinulosa*, *Halophila ovalis, Halodule uninervis*, *Zostera muelleri*, *Cymodocea serrulata*, and *Syringodium isoetifolium* ^19^.

### Georeferenced Photo Transects

Detailed information on seagrass species and percentage cover was gathered in the clear shallow waters of the Eastern Banks region of Moreton Bay using a repeatable and fine spatial scale (sampling every 2 m) technique for surveying benthic cover^20,21^. The technique requires a snorkeller manually capture georeferenced photographs along defined transects.

For the 2004 study, a 100 m transect tape was deployed at each transect start site at a maximum depth of 3.0 m. The snorkeller followed the tape and took a photograph of the benthos every 2.0 m. From 2007 onwards, while swimming, the snorkeller towed a standard handheld GPS (e.g. Garmin eTrex, Garmin 72) at the surface in a waterproof bag, to log each photograph’s position. This enabled accurate registration of the location of the acquisition of each photograph, which was assigned in the lab subsequently via time synchronization, with the track log from the towed GPS. Once this method was established, a transect tape was no longer deployed and transect lengths were extended to distances of 500–800 m. The start and end point of each transect was defined by GPS waypoints, permitting accurate revisits in subsequent years. The distance between successive photographs was estimated by the snorkeller’s kick cycle. Practically this meant that, as an example for 2007, the average interval distance was 4.2 m, ranging from 0.1 m to 12.9 m with a standard deviation of 1.6 m. Variability in the interval distance between photographs is present due to: the kick cycle of the different snorkellers acquiring the data, strength and direction of the ocean current, and, image capture failure. However this was not considered a problem as the exact location of each photograph was known through the GPS synchronisation.

Benthic photographs were captured with a standard digital camera in a waterproof housing (e.g. Sony Cyber shot, Canon AA540, or Lumix). Using a plumb-line attached to the camera, photographs were taken at as close to 0.5 m vertically from the bottom.

The locations of the transects were chosen to ensure they traversed gradients or edge features to detect any change in species or percent cover over these features. This was done initially through visual assessment of existing satellite imagery in combination with expert knowledge of the study area. The aim was to produce data that provided an adequate representation of the variety of seagrass species and coverage percentages observed across the Eastern Banks. Transects were revisited in subsequent surveys. Additional transects were included on subsequent trips based on increased knowledge of the environment. The full data set is available at pangaea.de DOI 10.1594/PANGAEA.846147.

### Photograph analysis for seagrass species and percentage cover

Species composition and percentage cover were determined for each photograph by assessing seagrass species or bottom type (e.g. algae, coral, sponge, sand etc.) at 24 randomly distributed points using Coral Point Count Excel® v4.0 (ref. 22). The categories were chosen to represent the variations in composition of seagrass species, seagrass health and alternative bottom constituents (Table 1 (available online only)).

## Data Records

Detailed information regarding the number of transects and benthic photographs acquired for each field campaign are documented in Table 2 and can be accessed online (Data citation 1). The DOI numbers for each individual data set are also provided.

## Technical Validation

To understand the validation technique applied to these data sets, it is important to reiterate the purpose of collecting the data set itself, which was: a fast field method to gather seagrass information over a large extent, whilst accurately representing variability in seagrass composition, for use as calibration data for mapping (2/3 points) and to validate the output classification (1/3 points). Validation of the data set was conducted on various levels, and included: correction of errant data point locations, standardisation of photo capture method and conditions, and benthic photograph analysis.

### Validation of data point position

The purpose of the field data was to align it in correct spatial context with the satellite imagery. Therefore, it was important that the satellite image to field data positional mis-registration was kept to a minimum. In this case, this was a maximum mis-registration of two pixels for imagery that had a pixel size of 2–4 m, dependant on the satellite sensor chosen. During the surveys a standard GPS (e.g. Garmin Map76) was used to determine the position of each photograph/data point with a known accuracy of 5–10 m and the satellite imagery was georeferenced against a base image to within one pixel^3^. The position of field data was validated against the georeferenced satellite imagery. This was done visually by overlaying data points on the imagery, then choosing points that covered defined benthic compositions in the field (e.g. 10 m diameter circle of seagrass species). If a data point was not aligned it was moved to best fit. The inherent size of the pixels in the imagery absorbed significant placement error.

### Standardisation of photograph capture

To standardise photograph capture for each field campaign, each camera and lens setup was calibrated prior to survey, so as to capture a footprint that covered the same extent of the benthos. This was accomplished by attaching a plumb-line to the camera system such that when it would touch the bottom, the captured photographs would represent ~1 m^2^ of the benthos. To do this standardisation, the camera was moved vertically over a marked 1 m^2^ until a photograph enveloped the entire 1 m^2^ area as closely as possible. The plumb-line was fixed at this point. During the survey the operator used the plumb-line to determine the camera height above the ground. When held vertically with the lead weight just touching the substrate this permitted reproducible capture of photographs that covered the same area for all surveys. Light conditions were generally the same for each field trip, the data collected over a consecutive 3–4 day period, with stable weather, water clarity conditions and tidal range. Ideally light conditions would have been standardised using a strobe, however this would slow down the speed of the transects and therefore the surveys.

### Derivation of benthic information from photographs

Validation of manual photograph analysis was accomplished several ways. Two authors analysed the photographs, Roelfsema (data for 2004 and 2007), and Kovacs (data for 2011 to 2014). One hundred random photographs were assessed by both operators and discrepancies were corrected through discussion of each individual photograph. Additionally, a photograph library with representative images of specific benthic categories was used to help standardise the analysis. As the field photographs are archived, it is possible to revisit the photographs to check the categories assigned or to check for new categories not previously considered important from a biological perspective (e.g. unknown disease or seagrass impact), or from a remote sensing classification perspective (e.g. dark versus light sand).

Following manual photograph analysis, the field photographs and corresponding data points were projected on satellite imagery and assessed visually for accuracy of composition. This provided additional validation:

- Supported by local expert knowledge of the authors e.g. *Cymodocea serrulata* occurs mostly in dense patches that appear as dark areas on the satellite image, and certain seagrass species do not occur in all places due to biological constraints. If based from the satellite imagery the category assigned was incorrect, the photograph(s) would be reassessed.
- As the generalised seagrass species distribution is the same over time, with fluctuations observed in percentage cover. Therefore, if a species was different for one data set but not others the photos were reassessed.
- As abrupt changes in benthic composition in successive photographs in a transect is uncommon. Hence, in situations where an abrupt change was observed, the data set would be checked. Thus, an internal control is provided as neighbouring data points provide validation of benthic composition.

An additional comparison was conducted between the seagrass data points with corresponding locations in the seagrass property maps created with the field data and satellite imagery. Errors in assigning categories during photograph analysis may influence the training of the classifier for map creation from the satellite imagery. Thus errors identified in the output maps may indicate errors in category assignment during photograph analysis and indicate that a photograph and category assignment review is in order.

## Additional Information

Table 1 is only available in the online version of this paper.

**How to cite this article:** Roelfsema, C. M. *et al.* Field data sets for seagrass biophysical properties for the Eastern Banks, Moreton Bay, Australia, 2004–2014. *Sci. Data*. 2:150040 doi: 10.1038/sdata.2015.40 (2015).

## Supplementary Material



## Figures and Tables

**Table 1:** Seagrass and bottom type categories and their descriptive codes used in the photograph analysis

**First Level (code)**	**Description**	**Second Level**	**Code**	**Description**
Seagrass (SG)	Not macroalgae; generally green	*Halodule uninervis*	SGHU	Narrow, strap like leaf (0.2 cm); leaf tip tri-dentate or pointed.
		*Zostera muelleri (eel grass)*	SGZM	Strap-like leaves 0.5 cm wide; leaf tip smooth and rounded; up to 30 cm long.
		*Cymodocea serrulata*	SGCS	Wide strap-like leaves (1 cm); leaf tip rounded; serrated edge.
		*Syringodium isoetifolium*	SGSI	Thin cylindrical like leaf (spaghetti); fragile.
		*Halophila ovalis*	SGHO	Paddle-like shape low to benthos; leaf margins smooth.
		*Halophila spinulosa*	SGHS	Compound leaf; leaflets serrated.
		Seagrass – Species Unknown	SGOT	Other seagrass species.
		Seagrass detritus	SGD	Dead seagrass floating around on the benthos.
Seagrass Plus (SGP)	Seagrass covered in epiphyte or algae, or, dark in colour.	*Halodule uninervis +* Algae	SGHUA	Like Halodule uninervis but covered with dense hairy, green, brown algae (macro or epiphytic).
		*Zostera muelleri + Dark*	SGZMD	Like Zostera muelleri but leaves look dark brown/black.
		*Zostera muelleri +* Algae	SGZMA	Like Zostera muelleri but covered with dense hairy, green, brown algae (macro or epiphytic).
		*Cymodocea serrulata +* Algae	SGCSA	Like Cymodocea serrulata but covered with dense hairy, green, brown algae (macro or epiphytic).
		*Syringodium isoetifolium + Epiphyte*	SGSIE	Like Syringodium isoetifolium but with grey particulate matter attached to the leaves.
		*Syringodium isoetifolium +* hairy algae	SGSIA	Like Syringodium isoetifolium but with dense hairy, green, brown, dark grey algae.
		*Halophila ovalis +* Algae	SGHOA	Like Halophila ovalis but covered with dense hairy, green, brown algae (macro or epiphytic).
		*Halophila spinulosa +* Algae	SGHSA	Like Halophila spinulosa but covered with dense hairy, green, brown algae (macro or epiphytic).
Macro Algae Calcareous (MAC)		*Padina sp.*	ACP	Fan-shaped algae; containing white calcareous concentric rings.
		*Halimeda sp.*	ACH	Calcified string of green scale-shaped segments.
		*Udotea sp.*	ACU	Individual calcified green fane-like segments.
		Macro Algae Calcareous—Genus Unknown	ACOT	Other MAC.
Macro Algae Non Calcareous (MA)	Not seagrass and major algae family on reef systems (Taxonomic based) which are not calcareous	*Sargassum sp.*	ASA	Attached to substrate; brown; vertical in the water column due to air trapped in leaf chambers.
		*Caulerpa sp.*	ACA	Green, grape-like; connected via branching structures over the substrate.
		*Hydroclatharus sp.*	AHY	Brown algae; uneven holes; reminiscent of a 'worn out' dish cloth.
		*Hydroclatharus sp*. on Seagrass	AHYS	As above.
		MA—Genus Unknown	AOT	Other MA.
Cyanobacteria and Other Algae (COA)		Microphytobenthic (MPB) on Sand	MPS	MPB on sand where sand patches are visible in the image.
		MPB-mat	MPM	MPB occupies complete image—no sand patches visible (100% cover).
		Macro Cyano on Sand	MCS	Hairy like strings of cyanobacteria on sand; generally longer than MPB or turf algae.
		Macro Cyano on other	MCO	Hairy like strings of cyanobacteria on anything other than sand.
Substratum (SU)	Bare substrate	Sand Light Colour	SSL	Will fall out of your hand; light colour.
		Sand Dark Colour	SSD	Will fall out of your hand; black, grey or brown.
Other (O)	Anything not a plant, algae or substrate	Sponge	OSP	Attached to the benthos; homogeneous colour, Often one colour with a small and a big hole.
		Urchin	OUR	Spiny and spherical. Can cause a negative impact if too many.
		Anemone	OAN	Sessile polyp attached to the benthos; array of tentacles.
		Pina (fan mussel)	OPI	Upright bivalve, 30-50cm long
		Small Shells	OSS	Discarded small shells, often very white in colour.
		Star Fish (Sea star)	OSF	Star-shaped, flat organism free to roam the benthos.
		Sea Cucumber	OSC	Elongated, cylindrical body, free to move around. Have commercial value.
		Rock	RO	Hard surface not included in any other category.
		Other	OOT	Other that is definitely worth noting, e.g. coral
		Other Dead	OD	Other live type which is now dead

**Table 2:** Overview of the data files and formats that represent the georeferenced photographs captured during each of the field campaigns

**Date**	**# transects**	**# photographs**	**Length of transect (m)**	**DOI (pangaea.de)**
2004 June (w)	56	2316	100	10.1594/PANGAEA.846264
2007 July (w)	16	1476	300-800	10.1594/PANGAEA.846142
2011 June (w)	35	4150	300-800	10.1594/PANGAEA.846143
2012 February (s)	30	3087	300-800	10.1594/PANGAEA.846144
2012 June (w)	32	3748	300-800	10.1594/PANGAEA.846146
2013 February (s)	30	3213	300-800	10.1594/PANGAEA.846185
2013 June (w)	30	4366	300-800	10.1594/PANGAEA.846186
2014 June (w)	39	4277	300-800	10.1594/PANGAEA.846266
(s=Australian summer, w=Australian winter; 2004 and 2007 analysed by Roelfsema, and 2011-2014 by Kovacs). The complete data set is available at Data citation 1.				
